# Selection of housekeeping genes for gene expression studies in human reticulocytes using real-time PCR

**DOI:** 10.1186/1471-2199-7-33

**Published:** 2006-10-06

**Authors:** Nicholas Silver, Steve Best, Jie Jiang, Swee Lay Thein

**Affiliations:** 1 Molecular Haematology, Division of Gene and Cell Based Therapy, King's College London School of Medicine at King's College Hospital, Denmark Hill, London, SE5 9PJ, UK

## Abstract

**Background:**

Control genes, which are often referred to as housekeeping genes, are frequently used to normalise mRNA levels between different samples. However, the expression level of these genes may vary among tissues or cells and may change under certain circumstances. Thus, the selection of housekeeping genes is critical for gene expression studies. To address this issue, 7 candidate housekeeping genes including several commonly used ones were investigated in isolated human reticulocytes. For this, a simple ΔCt approach was employed by comparing relative expression of 'pairs of genes' within each sample. On this basis, stability of the candidate housekeeping genes was ranked according to repeatability of the gene expression differences among 31 samples.

**Results:**

Initial screening of the expression pattern demonstrated that 1 of the 7 genes was expressed at very low levels in reticulocytes and was excluded from further analysis. The range of expression stability of the other 6 genes was (from most stable to least stable): *GAPDH *(glyceraldehyde 3-phosphate dehydrogenase), *SDHA *(succinate dehydrogenase), *HPRT1 *(hypoxanthine phosphoribosyl transferase 1), *HBS1L *(*HBS1*-like protein) and *AHSP *(alpha haemoglobin stabilising protein), followed by *B2M *(beta-2-microglobulin).

**Conclusion:**

Using this simple approach, *GAPDH *was found to be the most suitable housekeeping gene for expression studies in reticulocytes while the commonly used *B2M *should be avoided.

## Background

Reticulocytes are juvenile enucleated red cells produced during erythropoiesis and spend approximately 24 hours in the bone marrow before entering the peripheral circulation. Reticulocytes persist for a few days in the circulation before forming the slightly smaller, mature red blood cell at which time any residual RNA the reticulocytes still possessed, is lost [1]. Peripheral blood reticulocytes are not only readily accessible, but remnants of RNA they still harbour are likely to represent gene expression profiles of patients. This makes them potentially very useful in looking for subtle changes in gene expression, particularly for quantitative traits involved in the phenotypic outcome of haemoglobinopathies. In order to reach the sensitivity needed to detect such subtle changes in gene expression, quantitative real time PCR (Q-RT-PCR) was employed.

When comparing gene expression in different samples it is crucial to consider experimental variations such as amount of starting material, RNA extraction and reverse transcription efficiencies. To account for these, accuracy of Q-RT-PCR relies on normalisation to an internal control, often referred to as a housekeeping gene [2,3]

The prerequisite of a suitable housekeeping gene is that it should, of course, be adequately expressed in the tissue of interest, but most importantly, that it shows minimal variability in expression between samples and under the experimental conditions used [2,4]. A study carried out in 1999 by Suzuki *et al *[5] reported that over 90% of the RNA transcription analysis published in high impact journals used a single housekeeping gene, and this is still common practise today. It is abundantly clear, however, that many of these control genes can show unacceptable variability in expression [6-10]. Appropriate validation of housekeeping genes in any new experimental system is therefore crucial.

Various attempts to find the most appropriate method for the selection of housekeeping genes have been made. One software-based approach, developed by Vandesompele *et al *[11] involves the use of normalising to more than one housekeeping gene, where, very briefly, an algorithm based computer program, geNorm is used to determine the most stable control genes from a panel of candidate housekeeping genes via a stepwise exclusion or ranking process, and this is then followed by geometric averaging of a selection of the most stable control genes. Other software based approaches include BestKeeper [12], an Excel-based tool and Normfinder [13] an add-in for Microsoft Excel which adds the NormFinder functionality directly to the Excel software package. Although accurate and reproducible, these approaches require the use of specialist programs, and small quantities of starting material in some experiments can be prohibitive and as a result may not be appropriate for reticulocyte RNA. A further, more common strategy for the investigation of gene expression involves the standardisation of starting mRNA whereby a constant amount of RNA is added to each reverse transcription reaction [14]. In some instances, it is impossible to quantify this parameter, for example, for reticulocyte RNA, only minimal amounts of RNA are obtained from patient samples and errors in measurement can occur due to protein contamination in the RNA sample. We have adopted a method, similar to that described by Vandesompele *et al *[11], whereby 'pairs of genes' are compared using a simple ΔCt approach.

In this study, we chose to investigate a panel of 7 housekeeping genes (table 1), including those expressed in red blood cells and haemopoietic tissue. Furthermore, validation was carried out via normalisation of γ-globin to the highest scoring controls and then correlating γ-globin with the corresponding protein levels (fetal haemoglobin, α_2_β_2_)

## Results

### Expression profiling of housekeeping genes

**Figure 1:**
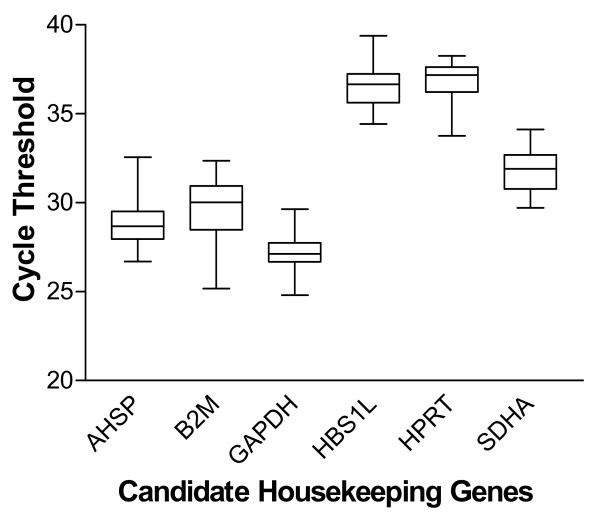
**Q-RT-PCR cycle threshold values for 7 candidate housekeeping genes among 31 adult human reticulocyte samples**. Candidate housekeeping genes include *AHSP*, *B2M*, *GAPDH*, *HBS1L*, *HPRT1 *and *SDHA*.

Primers were either designed or selected from 'off the shelf' for a total of 7 control genes – see table 2 for details. Particular attention was paid to selecting genes that belong to different functional classes, which significantly reduces the chance that genes might be co-regulated. In addition to K562 cells, the expression level of these 7 internal control genes was determined in 31 reticulocyte samples from 31 healthy volunteers (Figure 1). Note, in the expression profiling in the reticulocyte samples, *ACTB *showed equivalent levels of expression to NTCs (no template controls) and was therefore excluded from further studies.

### Q-RT-PCR efficiency and intra- and inter- assay variability

Quantitative real-time PCR was used to measure the RNA transcription level of various candidate housekeeping genes. To compare different RNA transcription levels, the Ct values were compared directly. The Ct is defined as the number of cycles needed for the fluorescence to reach a specific threshold level of detection and is inversely correlated with the amount of template nucleic acid present in the reaction [15]. To ensure comparability between the 7 PCR assays, efficiency of each individual assay was determined by measuring serial dilutions of 500 ng cDNA from K562 cells in triplicate. Inter-assay variation was investigated in three independent runs performed on three consecutive days. Only Ct values < 40 were used for calculation of the PCR efficiency from the given slope generated in the SDS 2.2 software according to the equation: PCR efficiency = (10^[-1/slope]^-1) × 100. All PCR assays displayed efficiency between 92% and 100%.

Intra-assay variation was < 0.67% and inter-assay variation < 0.4% for all assays.

### Approaches used for housekeeping gene selection ΔCt Approach

It is not always satisfactory to rely on the precision and reproducibility of total RNA standardisation alone for Q-RT-PCR applications, as a very stringent estimate of concentration is required. To bypass this potential source of error we used a similar technique to Vandesompele *et al*. However, without compromising accuracy, we kept the level of mathematical methodology to a minimum in order to allow non-specialist personnel to easily follow a similar route in housekeeping discovery. Here we employed a ΔCt method by comparing relative expression of 'pairs of genes' within each sample to confidently identify useful housekeeping genes. If the ΔCt value between the two genes remains constant when analysed in different samples of reticulocyte RNA, it means either both genes are stably expressed among those samples, or co-regulated (here we assume the stability of both genes). However, if the ΔCt fluctuates, then 1 or both genes are variably expressed. Introduction of a third, fourth, fifth gene into the comparisons will provide more information on which pairs show less variability and hence which gene(s) has stable expression among samples tested. These can then be ranked or discarded. Using this technique of 'process of elimination' means relatively large panels of genes can be compared against one another and either selected or eliminated on the basis of ΔCt. Ultimately, a housekeeping gene(s) that is appropriate to use in the experimental system of interest can be selected.

**Figure 2:**
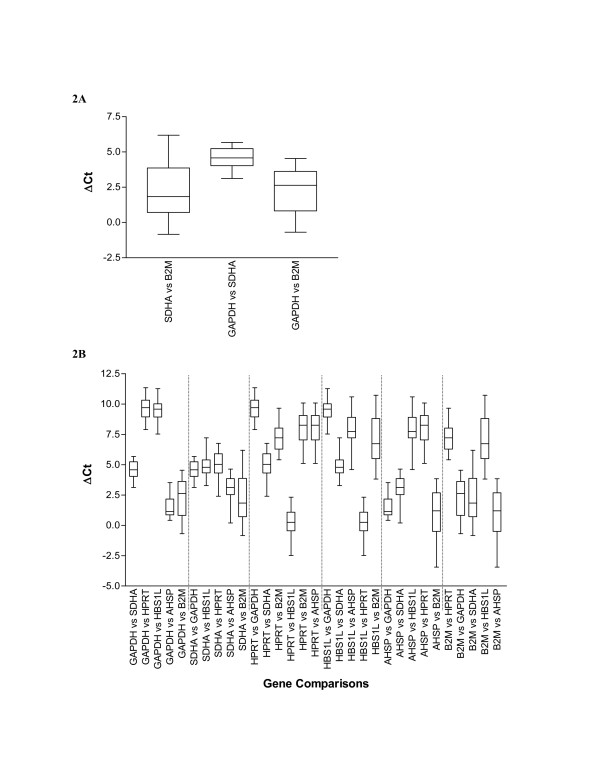
**ΔCt approach to housekeeping gene selection**. ΔCt variability in candidate housekeeping gene comparisons are shown as medians (lines), 25^th ^percentile to the 75^th ^percentile (boxes) and ranges (whiskers) for 31 human reticulocyte samples. **A **– highlights a subset of candidate housekeeping genes including *B2M*, *GAPDH *and *SDHA*. **B **– shows comparisons of the complete set of candidate housekeeping genes, which include *AHSP*, *B2M*, *GAPDH*, *HBS1L*, *HPRT1 *and *SDHA*.

Exampled in figure 2 is this ΔCt approach to housekeeping gene selection. Using just three of the genes (figure 2A) from the main housekeeping gene panel, *SDHA*, *B2M *and *GAPDH *for 31 samples, it can be seen that when *SDHA *and *B2M *are compared there is a relatively large deviation in the ΔCt values over the 31 samples, indicating that one or both genes are variable. When *SDHA *is then compared to *GAPDH *the deviation in ΔCt values among the 31 samples falls considerably, indicating that both *SDHA *and *GAPDH *show relatively stable expression. However, once *B2M *is re-introduced into the comparisons against *GAPDH*, the standard deviation of ΔCt can again be seen to increase and hence it can be concluded that *B2M *is the gene displaying variable expression between samples and can then be eliminated or given a low ranking.

### Relative Stability of housekeeping genes

Gene expression levels were measured by Q-RT-PCR and expression stabilities evaluated via the ΔCt method and standard deviation. Taking all the genes into account and by comparing all possible gene combinations (figure 2B, table 3), a pattern forms whereby genes tend to be associated with either increased or decreased levels of deviation in ΔCt among the 31 reticulocyte samples, and hence, either an increase or decease in the level of variability in gene expression. When *GAPDH *and *SDHA *are compared against the other 5 genes in their respective gene panels, they tend to be associated with the least amount of deviation (average StdDev of 0.99 and 1.10 respectively) and hence least amount of variability. Whereas, when *B2M *is compared against the other 5 genes in the panel there is a considerable increase in deviation (average StdDev 1.64) indicating an increase in variability. *HPRT1*, *HBS1L *and *AHSP *all show an intermediate level of deviation (average StdDev of 1.13, 1.23 and 1.30 respectively) and hence an intermediate level of variability. Overall rankings are as follows (figure 2B, table 3): *GAPDH*, *SDHA*, *HPRT1*, *HBS1L*, *AHSP*, followed by *B2M*.

Based on the expression stability, the data suggests that *GAPDH *would be the most suitable housekeeping gene to use in reticulocyte studies. A popular housekeeper, *B2M*, should be avoided. In addition, comparison of *GAPDH *against the other 5 genes in the gene panel not only indicates its level of stability but also shows this level of stability cannot be due to co-regulation as each gene has a different function.

### Evaluation and validation of selected candidate housekeeping genes using γ-globin

Following identification of the most stable housekeeping genes from the full gene panel of 7 genes, a method was needed for their validation. This validation process was based on protein (Hb F, α_2_γ_2_) levels as measured by high performance liquid chromatography (HPLC, BioRad Variant).

**Figure 3:**
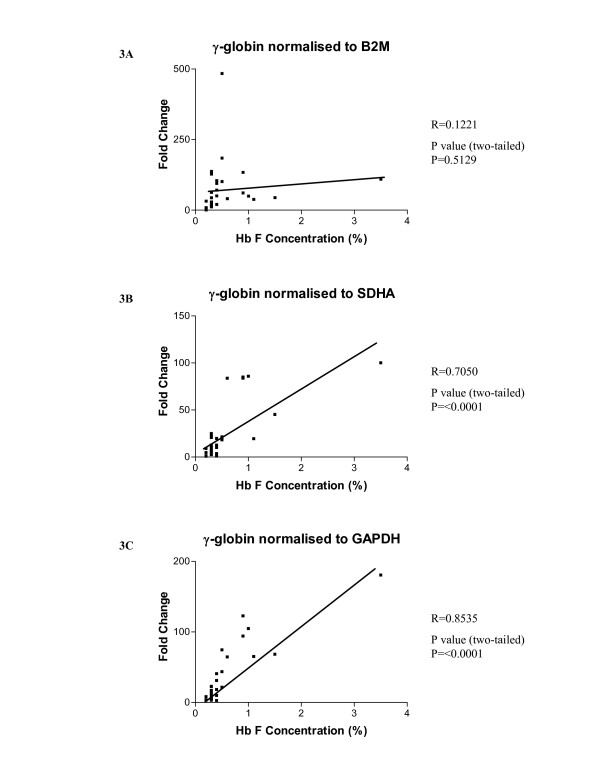
**Housekeeping validation**: The above plots show the relationship between comparative Ct for γ-globin normalised to a) *B2M*, b) *SDHA *and c) *GAPDH *and Hb F. The ideal scenario is for the γ-globin vs Hb F correlation to improve as progressively better housekeeping genes are used.

We expect a tight correlation between levels of Hb F and γ-globin expression and consequently, correlation of γ-globin mRNA and the corresponding levels of Hb F would not hold if γ-globin expression were normalised to an inappropriate housekeeping gene. Figures 3A, C demonstrate this.

As seen in figure 3A, when γ-globin is normalised to *B2M *no relationship can be seen (R = 0.1221, P value (two-tailed) 0.5129), indicating *B2M *inappropriate as a housekeeping gene. However, there is a considerable improvement in the correlation when *SDHA *is used for normalisation; *SDHA *R = 0.7050, P value (two-tailed) P < 0.0001. The third gene exampled (figure 3C) in this validation process was *GAPDH*, which improves the correlation even further between γ-globin and Hb F; R = 0.8535, P value (two-tailed) P < 0.0001.

## Discussion

Reticulocytes are present in small numbers in peripheral blood, can be readily isolated, and still harbour remnants of genetic material that likely represent gene expression profiles of patients. This makes them a good source of biological material in which to study differential gene expression. Quantitative trait loci involved in the phenotypic outcome of haemoglobinopathies, which are mapped and localised by linkage and association studies, usually exert their effect through subtle changes in gene expression. To detect these subtle changes, a sensitive method of detection is needed. Real time PCR is one of the most sensitive and reproducible quantification methods for gene expression analysis. It provides simultaneous measurement of gene expression in many different samples for a number of genes. However, many different factors in real-time PCR may effect the results, including the selection of the housekeeping genes. The 'ideal' housekeeping gene should be constantly transcribed in all cell types and tissues and remain stable between samples taken from different time points and under different experimental conditions. However, it is impossible to find a 'universal' housekeeping gene having stable expression under all these conditions [4,16]. For example, *ACTB*, *GAPDH *and 18S are the most commonly used housekeeping genes, but a number of studies have provided evidence that their transcription levels vary considerably between different individuals, different cell types, different developmental stages and under different experimental conditions [4,5,9]. Therefore, for accurate analysis of gene expression, thorough validation of candidate housekeeping genes is crucial.

Another crucial factor in gene expression analysis concerns using the most appropriate method for housekeeping gene selection. A significant consideration when it comes to this is the small quantities of mRNA obtained from reticulocytes. Attempts to find the most appropriate method has involved an approach developed by Vandesompele *et al *[11]. This approach relies on the principle that two perfect housekeeping genes would be identical in all samples in all experimental conditions or cell types. Variation in expression ratios between different samples reflects the fact that one or both of the genes are not stably expressed. Therefore, increasing variation in this ratio corresponds to decreasing expression stability. A visual basic application for Microsoft Excel – termed geNorm – has been written that uses an algorithm to calculate M, a gene expression stability measure, which is the mean pairwise variation for a gene compared with all other tested control genes. Genes with higher M values have greater variation in expression, and via a stepwise exclusion process genes can be ranked. For an accurate measure of expression levels, normalisation by multiple housekeeping genes is suggested. Consequently, a normalisation factor based on the expression levels of the best performing housekeeping genes must be calculated via averaging of the control genes using the geometric mean. It is suggested that 3 stable control genes should suffice for accurate normalisation of samples with relatively low expression variation, whereas other tissue panels require a fourth, or even a fifth control gene to capture the variation. Further software based approaches include BestKeeper [12] and Normfinder [13]. BestKeeper, an Excel-based tool also uses 'pair-wise' correlations, and can determine the best suited standards, out of 10 candidates, and combine them into an index. Where the earlier presented geNorm software is restricted to housekeeping gene analysis only, the index generated by BestKeeper can then be compared with a further 10 genes to decide whether they are differentially expressed, for example, under an applied treatment. Here, all data processing is based on crossing points [12]. Normfinder, whose strategy is rooted in a mathematical model of gene expression, enables estimation not only of the overall variation of the candidate normalisation genes but also of the variation between sample subgroups of the sample set. Notably, the strategy provides a direct measure for the expression variation, enabling the user to evaluate the systematic error introduced when using the gene. It has also been reported to show relatively low sensitivity towards coregulation of the candidate normalisation genes [13]. Although very accurate, these methods rely on the design of specialist software programs, and where the use of multiple housekeeping genes are suggested, it is difficult to obtain enough reticulocyte sample to realistically use 3 or more housekeeping genes for normalisation. A further common strategy for the investigation of gene expression involves the standardisation of starting mRNA whereby a constant amount of total RNA is added to each reverse transcription reaction [14]. In some instances however, it is impossible to quantify this parameter, for example, because only minimal amounts of RNA are obtained from patient samples, errors in measurement can occur due to protein contamination in the RNA sample. We have therefore adopted a ΔCt approach; a method similar to that described by Vandesompele *et al*, whereby 'pairs of genes' are compared. This simple ΔCt approach bypasses the need to accurately quantify input RNA and instead uses ΔCt comparisons between genes.

Results show that the level of RNA transcription for some of the candidate housekeeping genes varied considerably and in some cases RNA transcription levels were too low to quantify accurately or reproducibly. This was particularly the case with *ACTB*, and this would therefore be completely inappropriate to use as a housekeeping gene. Out of all the genes tested, those that scored highest in their requirements as housekeeping genes were *GAPDH*, *SDHA *and *HPRT1*. However, when carrying out ΔCt comparisons, in order to provide the highest level of accuracy, the Ct values compared should not be vastly different. *HPRT1 *expression was found to be relatively low and it would therefore be inappropriate to compare this against a relatively highly expressed gene, for example, against γ-globin. *GAPDH *fulfilled most criteria as a suitable housekeeping gene in that it was strongly expressed, displayed minimal fluctuation and is likely to be independent of genes (i.e. not co-ordinately expressed) commonly involved in reticulocyte studies. In addition, *B2M*, which has been found to be a stable housekeeping gene in other biological systems [14,17] showed one of the highest levels of variation in this tissue. To confirm the validity of the approach used in the housekeeping gene selection process, γ-globin levels normalised against the gene panel were compared with their corresponding protein level. The strongest correlation was observed between *GAPDH*-normalised γ-globin expression and HPLC-measured Hb F levels, supporting *GAPDH *as an appropriate housekeeping gene for reticulocytes. The second highest ranking housekeeping gene from the gene panel was *SDHA*; *SDHA*-normalised γ-globin expression was then correlated with Hb F levels.

A large number of studies have been carried out concerning the validation of housekeeping genes in many different tissues and cell types. However, it has been difficult to find information on appropriate housekeeping genes for use in reticulocytes. To our knowledge, the only reported gene for normalisation in reticulocytes is *RPS19 *[18]. For accurate quantitation of a target gene, it is recommended that the housekeeper should have similar expression levels. This increases the sensitivity to detect subtle differences in gene expression. However, *RPS19 *is very highly expressed, as are many of the ribosomal genes, and would therefore be more appropriate for normalising very highly expressed genes such as α and β globins, but preferably not for genes with lower expression. Reticulocytes are often used in haematological studies and as a result, this information on alternative housekeeping genes may be very useful, if not essential for any future studies carried out involving this cell type.

## Conclusion

Peripheral reticulocytes are a readily accessible source of biological material for gene expression studies. For this reason, a survey of a small panel of genes including some of the more popular housekeeping genes was carried out. This was done using a simple ΔCt method based on comparing pairs of genes. We found at least one gene, *GAPDH*, which shows a good level of expression and stability. *ACTB *showed background levels of expression, and *B2M *was the least stable.

## Methods

### Samples

Clinical samples were collected from healthy unrelated adult volunteers (36 to 73 years old, median age 56; 5 male and 26 female) recruited through the St Thomas' UK Twin Registry [19]. This entailed collection of venous blood in both heparinized and EDTA-containing BD vacutainers™. Informed consent was obtained from all participants prior to collection. All donors had normal haemoglobin levels and normal RBC indices. Fetal haemoglobin (Hb F, α_2_γ_2_) percentages were measured by HPLC (Bio-Rad Laboratories, Inc., Hercules, CA). The study was approved by the Research Ethics Committee of St Thomas' Hospital, London, UK (E04/015).

### Isolation of peripheral blood reticulocyte RNA and reverse transcription

Reticulocytes were isolated from 10 ml of peripheral blood in heparin following a process of leukodepletion [20]. In brief, the reticulocyte-enriched, leukocyte-depleted red cell eluate was centrifuged to recover the reticulocytes which were then resuspended in 2 ml of cold PBS and 5 ml of TRI reagent (T-9424, Sigma-Aldrich) and stored at -80°C until RNA extraction.

RNA extraction was carried out on the thawed samples according to the manufacturer's protocol. The precipitated RNA was resuspended in 50 μl DEPC water and 1 microgram of RNA was reverse transcribed with 200U SuperScript™ III Reverse Transcriptase (Invitrogen) for 60 min at 50°C using Oligo-dT in a 20 μl volume.

Purity of reticulocytes, assessed by a Sysmex automated blood cell analyser, was 1 leukocyte per 35.5 × 10^3 ^RBCs and no detectable platelets.

### Selection of primers and probes

Primer and probe sequences are shown in table 2. These were synthesised by Applied Biosystems and included both customised primer and probe sets, designed via the Applied Biosystems Primer Express™ 2.0 software as well as pre-designed, gene-specific TaqMan^® ^probe and primer sets (TaqMan^® ^Gene Expression Assays, Applied Biosystems). Primers were used at 300 nM and probes at 200 nM in a 25 μl reaction. A selection of housekeeping genes were chosen, paying close attention to selecting genes that belong to different functional classes. This was aided by information provided by the human gene expression index database (HuGE) [21]. This was used to assess expression, variability and reproducibility of potential housekeeping genes.

### Real-Time quantitative RT-PCR

PCR was performed using the ABI Prism^® ^7900HT Sequence Detection System (Applied Biosystems) in 96 well microtitre plates using a final volume of 25μl. Amplifications were performed starting with a 2 min activation step for AmparaseUNG at 50°C, 10 min template denaturation step at 95°C, followed by 40 cycles of 95°C for 15 s and 60°C for 1 min.

## Abbreviations

*ACTB*, β-actin; *AHSP*, alpha haemoglobin stabilising protein; *B2M*, beta-2-microglobulin; cDNA, complementary DNA; ΔCt (dCt), delta cycle threshold; *HBG2*, γ-globin; *GAPDH*, glyceraldehyde 3-phosphate dehydrogenase; Hb F, haemoglobin F (fetal haemoglobin); *HBS1L*, *HBS1*-like protein; *HPRT1*, hypoxanthine-guanine phosphoribosyltransferase; mRNA, messenger RNA; NTC, no template control; PCR, polymerase chain reaction; Q-RT-PCR, quantitative real-time PCR; *SDHA*, succinate dehydrogenase; StdDev, standard deviation

## Authors' contributions

NS performed all the experimental procedures and was the primary author of the manuscript. SB participated in the study design. JJ supplied extracted mRNA from K562 cells ultimately used to provide cDNA for the generation of standard curves. All authors participated in the structuring and editing of the manuscript.

## Figures and Tables

**Table 1:** Panel of 7 candidate housekeeping genes selected and *HBG2*.

Gene symbol	mRNA accession number	Gene Name	Function	Gene aliases
*ACTB*	NM_001101	Beta-actin (β-actin)	Cytoskeletal structural protein	
*AHSP*	NM_016633	Alpha haemoglobin stabilising factor	Chaperone for human alpha globin	*ERAF, EDRF*
*B2M*	NM_004048	Beta-2-microglobulin	Cytoskeletal protein involved in cell locomotion	
*GAPDH*	NM_002046	Glyceraldehyde-3-phosphate dehydrogenase	Glycolytic enzyme	*G3PD, GAPD*
*HBS1L*	NM_006620	HBS1-like protein	Uncharacterised	*ERFS, HBS1*
*HPRT1*	NM_000194	Hypoxanthine guanine phosphoribosyl transferase I	Involved in the metabolic salvage of purines in mammals	*HPRT, HGPRT*
*SDHA*	NM_004168	Succinate dehydrogenase complex, subunit A, flavoprotein	Involved in the oxidation of succinate	*FP, SDH2, SDHF*
*HBG2*	NM_000184	Gamma globin (γ-globin)	Oxygen binding and transport	

**Table 2:** Sequence information for primers and probes used.

Gene	Oligo	Exon boundary	Sequence – 5' to 3'
*ACTB*			Assay by design (ABI)
			
AHSP	*AHSP *Forward	2 to 3	CTGAATCAGCAGGTCTTCAATGATC
	*AHSP *Reverse		GTCCCTGTACTTGGCCAGGA
	*AHSP *Probe		FAM-TCGTCTCTGAAGAAGACATGGTGACTGTGG -TAMRA
			
*B2M*	*B2M *Forward		GAGTATGCCTGCCGTGTG
	*B2M *Reverse		AATCCAAATGCGGCATCT
	*B2M *Probe	2 to 4	FAM-CCTCCATGATGCTGCTTACATGTCTC-TAMRA
			
*HBG2*	*HBG2 *Forward		CTGCACTGTGACAAGCTGCAT
	*HBG2 *Reverse		CGAAATGGATTGCCAAAACG
	*HBG2 *Probe	2 to 3	FAM-CCAGCACATTTCCCAGGAGCTTGAAGT-MGB
			
*GAPDH*			Assay by design (ABI)
			
*HBS1L*	*HBS1L *Forward		AAGGACAAGAATGAGGCAACAGTAT
	*HBS1L *Reverse		TTTGGCACAATTTCAGATTCACTT
	*HBS1L *Probe	4 to 5	FAM-AGCAAAAGGAAAACCAGTAGATTCCCAGACATC-TAMRA
			
*HPRT1*			Assay by design (ABI)
			
*SDHA*			Assay by design (ABI)

*AHSP *(alpha haemoglobin stabilising protein), *B2M *(beta-2-microglobulin), *HBG2 *(γ-globin), *GAPDH *(glyceraldehyde 3-phosphate dehydrogenase), *HBS1L *(*HBS1*-like protein), *HPRT *(hypoxanthine-guanine phosphoribosyltransferase), and *SDHA *(succinate dehydrogenase).

**Table 3:** Candidate housekeeping gene comparisons.

**Sample**	**MeanΔCt**	**StdDev**	**Mean StdDev**
*GAPDH *vs *SDHA*	4.54	0.71	
*GAPDH *vs *HPRT1*	9.58	0.90	
*GAPDH *vs *HBS1L*	9.44	0.91	
*GAPDH *vs *AHSP*	1.57	0.91	
*GAPDH *vs *B2M*	2.37	1.50	0.99
			
*SDHA *vs *GAPDH*	4.54	0.71	
*SDHA *vs *HBS1L*	4.9	0.96	
*SDHA *vs *HPRT1*	5.03	1.03	
*SDHA *vs *AHSP*	2.97	1.04	
*SDHA *vs *B2M*	2.18	1.77	1.10
			
*HPRT1 *vs *GAPDH*	9.58	0.90	
*HPRT1 *vs *SDHA*	5.03	1.03	
*HPRT1 *vs *B2M*	7.21	1.13	
*HPRT1 *vs *HBS1L*	0.13	1.23	
*HPRT1 *vs *AHSP*	8.00	1.35	1.13
			
*HBS1L *vs *GAPDH*	9.44	0.91	
*HBS1L *vs *SDHA*	4.9	0.96	
*HBS1L *vs *AHSP*	7.87	1.21	
*HBS1L *vs *HPRT1*	0.13	1.23	
*HBS1L *vs *B2M*	7.08	1.84	1.23
			
*AHSP *vs *GAPDH*	1.57	0.91	
*AHSP *vs *SDHA*	2.97	1.04	
*AHSP *vs *HBS1L*	7.87	1.21	
*AHSP *vs *HPRT1*	8.00	1.35	
*AHSP *vs *B2M*	0.79	1.98	1.30
			
*B2M *vs *HPRT1*	7.21	1.13	
*B2M *vs *GAPDH*	2.37	1.50	
*B2M *vs *SDHA*	2.18	1.77	
*B2M *vs *HBS1L*	7.08	1.84	
*B2M *vs *AHSP*	0.79	1.98	1.64

Mean ΔCt values are given for the mean difference between the genes over the 31 samples. Standard deviation (StdDev) is given for the variation in Ct values over the 31 samples.
